# Supplementary material for: Combined PARP and Dual Topoisomerase Inhibition Potentiates Genome Instability and Cell Death in Ovarian Cancer
Source: Int J Mol Sci. 2022 Sep 10;23(18):10503. doi: 10.3390/ijms231810503 (PMC9505822; doi:10.3390/ijms231810503)
Supplement: Supplementary file 1 [file ijms-23-10503-s001.zip › ijms-1903189-supplementary.pdf]

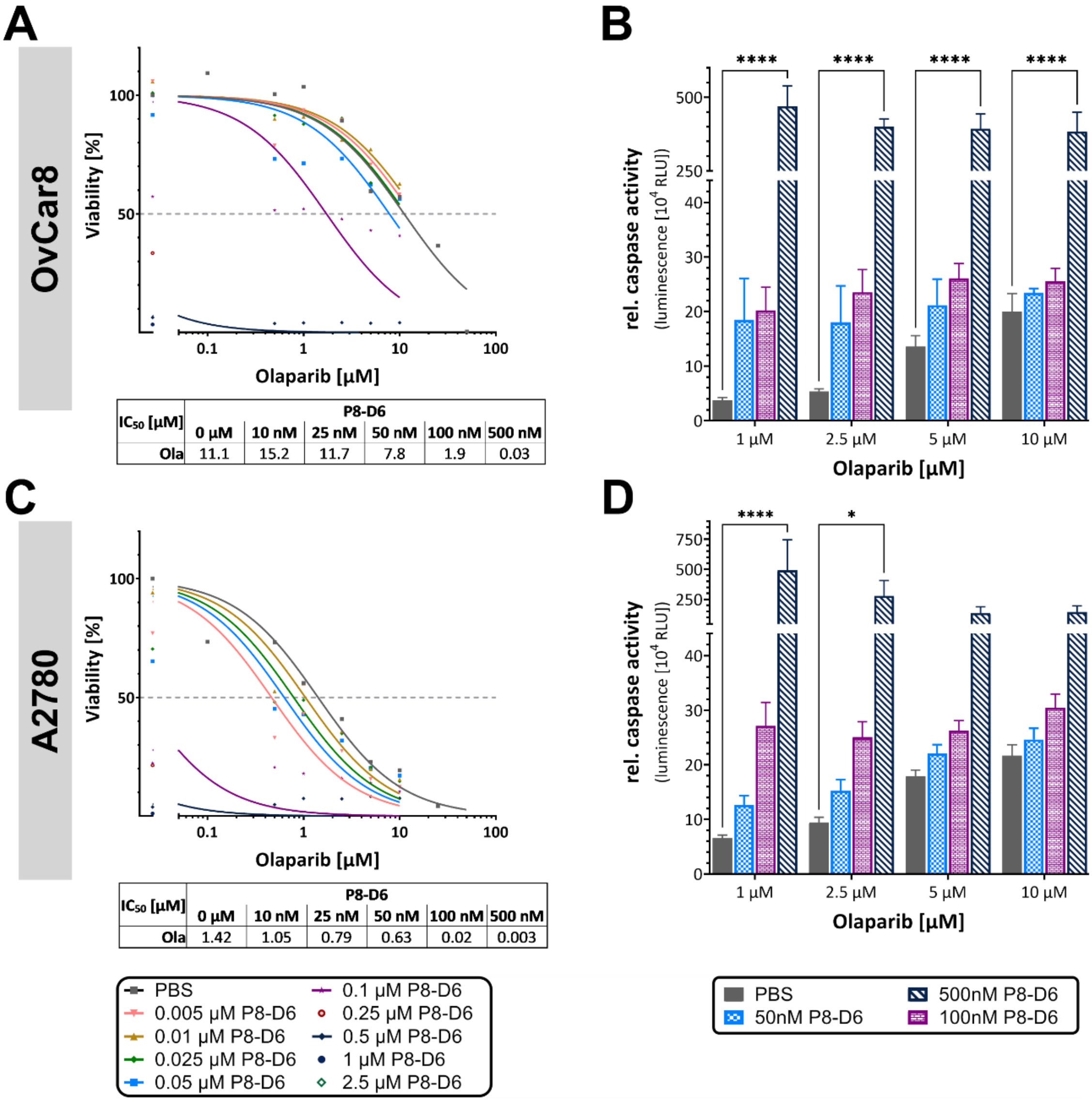

IC50 [µM]	P8-D6					
	0 µM	10 nM	25 nM	50 nM	100 nM	500 nM
Ola	11.1	15.2	11.7	7.8	1.9	0.03

IC50 [µM]	P8-D6					
	0 µM	10 nM	25 nM	50 nM	100 nM	500 nM
Ola	1.42	1.05	0.79	0.63	0.02	0.003

Figure S1. Antitumor benefit by adding P8-D6 to olaparib treatment in OC 2D culture. OvCar8 and A2780 cells were treated in 2D monolayer cell culture with different concentrations of olaparib and P8-D6 in single and combination therapy. Subsequently, the viability and caspase activity were determined. (A, C) The IC_{50} value of each P8-D6 concentration was calculated by using the viability data. (B, D) The apoptosis is represented as relative caspase activity. Data are means + SEM one-way ANOVA, * ($p < 0.05$), ** ($p < 0.01$), *** ($p < 0.001$), **** ($p < 0.0001$).

A

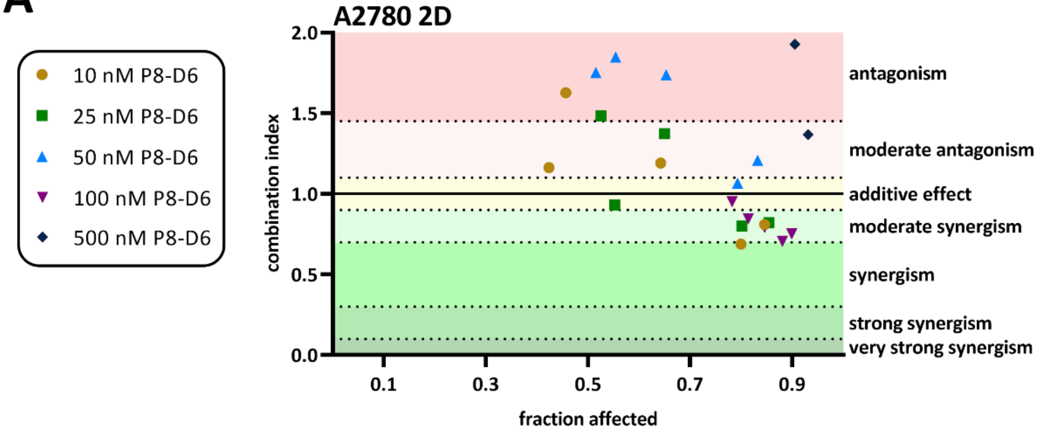

B

Combination therapy P8-D6 /Olaparib[µM]		fa	Monotherapy [µM]		Dose-Reduction index	
			P8-D6	Olaparib	P8-D6	Olaparib
0.005	0.5	0.463	0.03	0.79	6.4	1.58
0.01	0.5	0.424	0.03	0.62	2.78	1.25
0.025	0.5	0.552	0.04	1.37	1.76	2.75
0.05	0.5	0.515	0.04	1.09	0.77	2.19
0.1	0.5	0.782	0.11	7	1.14	13.99
0.5	0.5	0.931	0.37	52.67	0.74	105.34
0.005	1	0.512	0.04	1.07	7.62	1.07
0.01	1	0.457	0.03	0.76	3.14	0.76
0.025	1	0.526	0.04	1.17	1.6	1.17
0.05	1	0.554	0.04	1.39	0.89	1.39
0.1	1	0.814	0.14	9.42	1.35	9.42
0.5	1	0.898	0.25	27.48	0.5	27.48
0.005	2.5	0.666	0.07	2.85	13.49	1.14
0.01	2.5	0.643	0.06	2.43	6.15	0.97
0.025	2.5	0.650	0.06	2.56	2.53	1.02
0.05	2.5	0.653	0.06	2.61	1.28	1.04
0.1	2.5	0.846	0.17	13.35	1.66	5.34
0.5	2.5	0.905	0.27	31.02	0.54	12.41
0.005	5	0.833	0.15	11.47	30.34	2.29
0.01	5	0.800	0.12	8.21	12.49	1.64
0.025	5	0.801	0.13	8.32	5.03	1.66
0.05	5	0.793	0.12	7.69	2.4	1.54
0.1	5	0.881	0.22	20.89	2.15	4.18
0.5	5	0.902	0.26	29.16	0.52	5.83
0.005	10	0.877	0.21	19.83	41.73	1.98
0.01	10	0.846	0.17	13.35	16.57	1.34
0.025	10	0.854	0.18	14.75	7.02	1.47
0.05	10	0.832	0.15	11.41	3.02	1.14
0.1	10	0.899	0.25	27.87	2.54	2.79
0.5	10	0.911	0.29	34.45	0.58	3.45

DRI >10	Dose reduction
DRI >5	
DRI >1	
DRI < 1	Dose increase

Figure S2. Combination index (CI) analysis of olaparib in combination with P8-D6 in A2780 cells. (A) Combination index (CI) for drug combinations by olaparib and P8-D6 in A2780 cells. CI values computed according by CompuSyn software with viability values. Combinations were considered synergistic when CIs were below 1.0. The fraction affected (fa)-value represents the fraction of cell viability affected by therapy. (B) The monotherapy column defines the concentrations that are needed in monotherapy to affect a certain fraction of cells by therapy. DRI values represents the order of magnitude (fold) of dose reduction in combination setting compared with each drug alone.

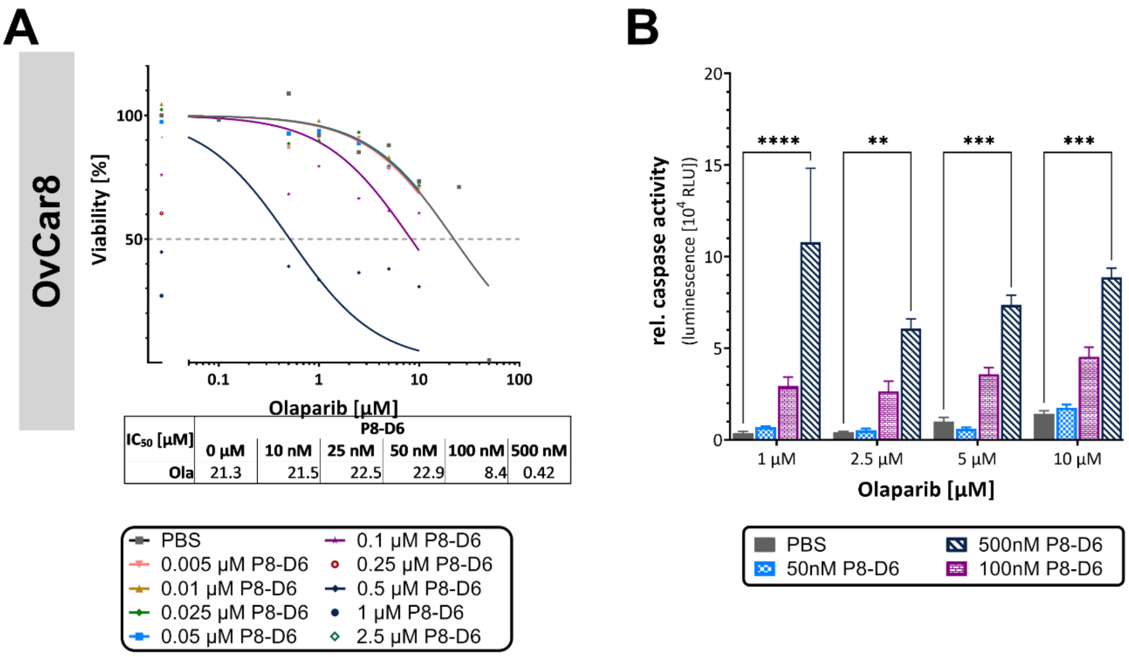

IC50 [µM]	P8-D6					
	0 µM	10 nM	25 nM	50 nM	100 nM	500 nM
Ola	21.3	21.5	22.5	22.9	8.4	0.42

Figure S3. Antitumor benefit by adding P8-D6 to olaparib treatment in OC 3D culture. OvCar8 and A2780 cells were treated in 2D monolayer cell culture with different concentrations of olaparib and P8-D6 in single and combination therapy. Subsequently, the viability and caspase activity were determined. (A, C) The IC_{50} value of each P8-D6 concentration was calculated by using the viability data. (B, D) The apoptosis is represented as relative caspase activity. Data are means + SEM one-way ANOVA, * (p <0.05), ** (p <0.01), *** (p <0.001), **** (p <0.0001).

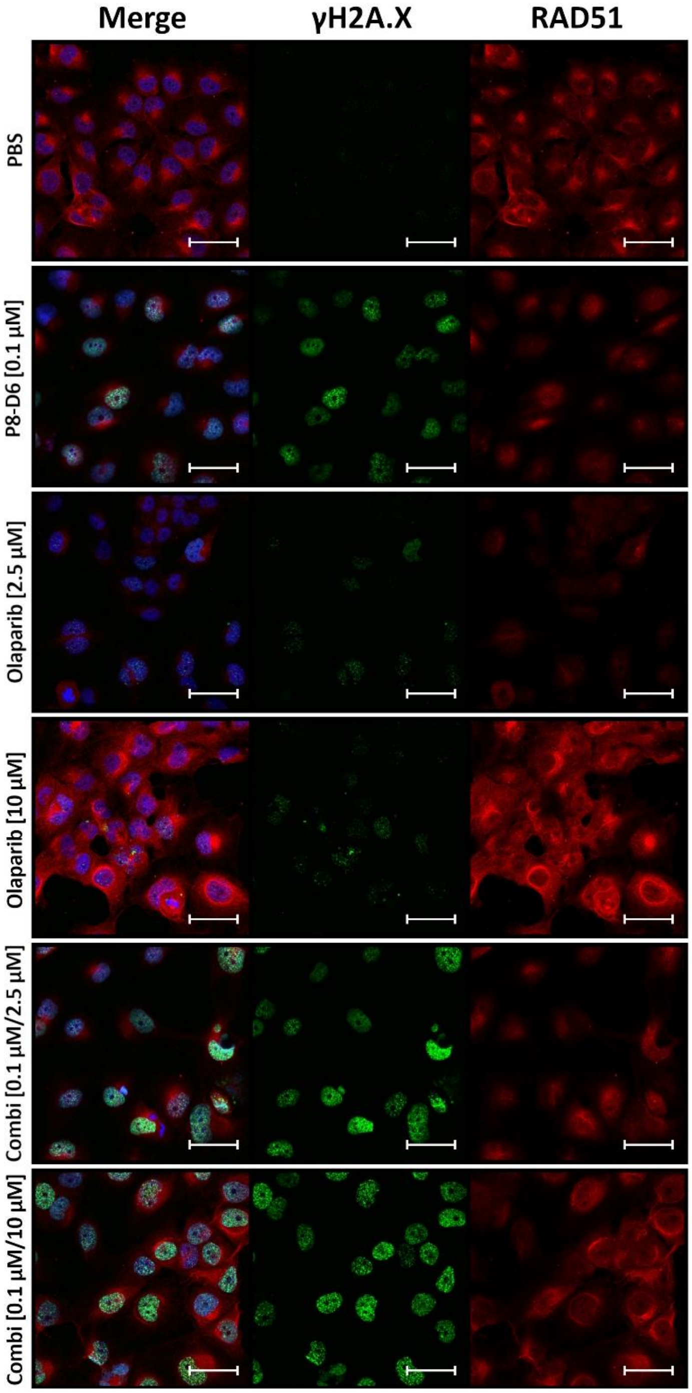

Figure S4. Olaparib potentiates γH2A.X and RAD51 recruitment for strand break induced by P8-D6 in OvCar8. Immunofluorescence images of OvCar8 2D monolayer: DAPI-stained nuclei (blue), γH2A.X (green), and RAD51 (red) after treatment with olaparib and/or P8-D6; scale bars, 50 µm.

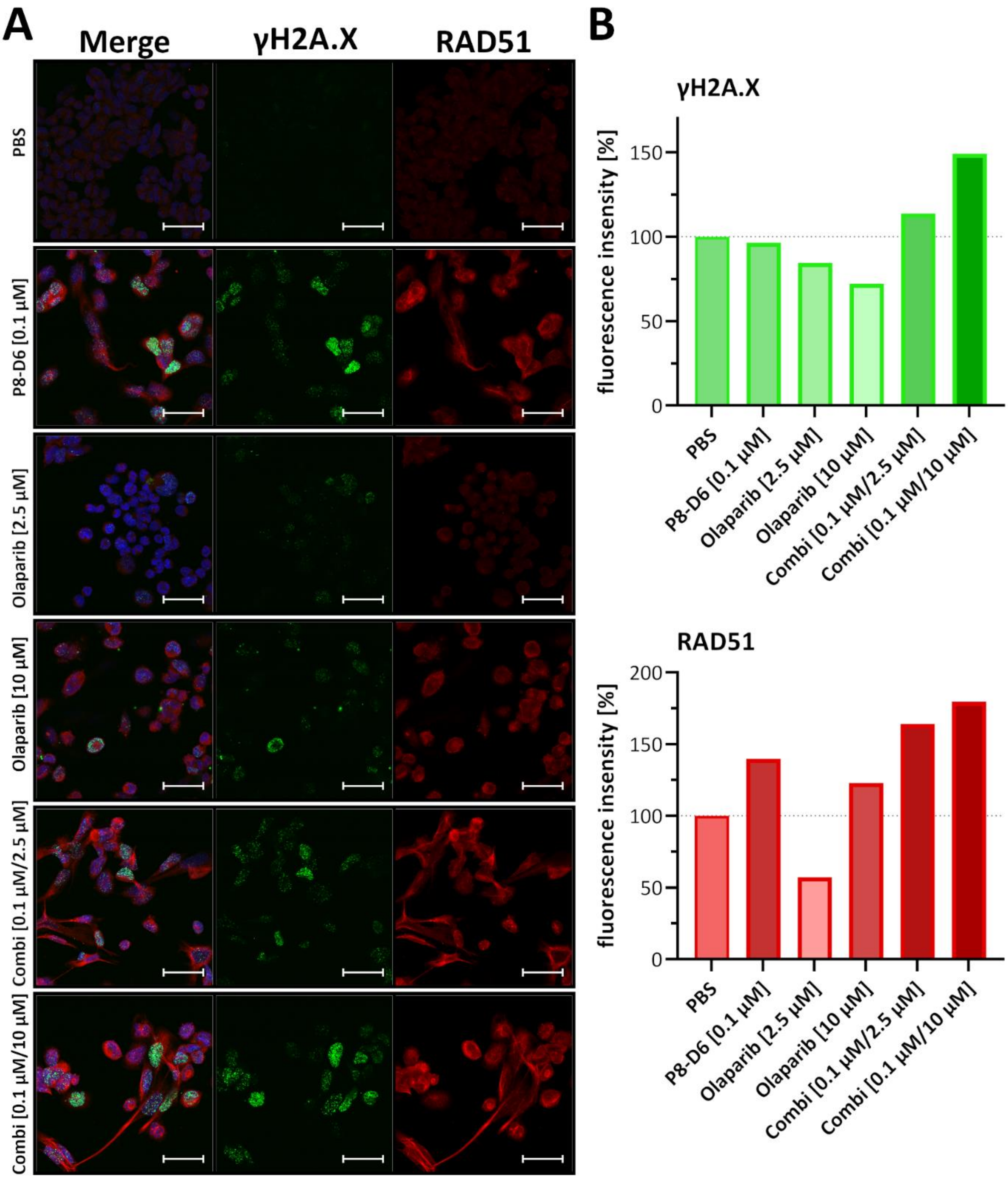

Figure S5. Olaparib potentiates γH2A.X and RAD51 recruitment for strand break induced by P8-D6 in A2780. (A) Immunofluorescence images of A2780 2D monolayer: DAPI-stained nuclei (blue), γH2A.X (green), and RAD51 (red) after treatment with olaparib and/or P8-D6; scale bars, 50 µm. (B) The intensity of the γH2A.X and RAD51 signal in relation to DAPI in A2780 2D monolayer after treatment.

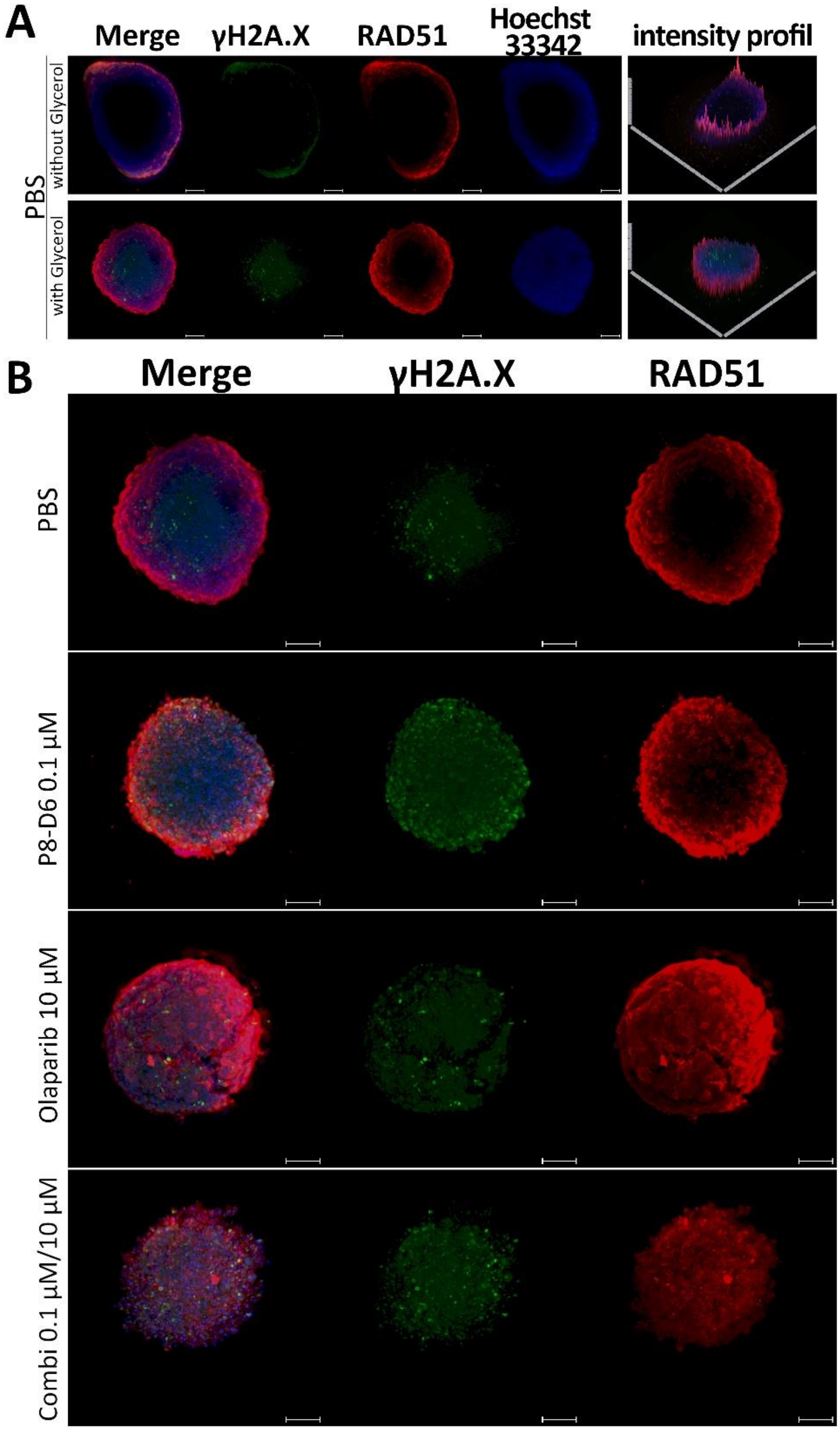

Figure S6. Optical transparency and induction of DNA-strand break in spheroids. (A) Optical transparency and detection of fluorescence signal intensity is depending on optical clearing protocol. Immunofluorescence images of OvCar8 spheroids treated with PBS and cleared with or without glycerol: Hoechst 33342-stained nuclei (blue), γH2A.X (green), and RAD51 (red). Intensity profil shows transparency potential of the spheroids; scale bars, 100 µm. (B) Confocal fluorescence z-stack images of OvCar8 spheroids were obtained with immunostaining against γH2A.X, RAD51 and Hoechst 33342. Spheroids were previously treated with olaparib and/or P8-D6 following antibodies staining with glycerol; scale bars, 100 µm
